# Spontaneous perforation of pyometra—is hysterectomy required in the emergent setting? A case report and literature review

**DOI:** 10.1093/jscr/rjac388

**Published:** 2022-08-30

**Authors:** Ikennah L Browne

**Affiliations:** Department of Surgery, University of Calgary, Cumming School of Medicine, Calgary, Alberta, Canada

## Abstract

Spontaneous perforation of pyometra is a rare event associated with significant morbidity and mortality when diffuse peritonitis is present. While malignant lesions of the cervical tract are the most common cause of pyometra, several benign conditions can contribute to this diagnosis. Traditionally hysterectomy has been the surgical approach of choice for this clinical entity; however, in the setting of septic shock, temporizing techniques may offer the opportunity to stabilize patients and complete a thorough work up before committing to definitive resection. This report explores a case of septic shock secondary to spontaneous perforation of pyometra that was definitively managed with peritoneal lavage and wide drainage. Intraoperative hysteroscopy and uterine biopsy were performed, and no malignancy was identified on final pathology. Intraoperative hysteroscopy along with peritoneal lavage and wide drainage may reduce the morbidity and mortality associated with sepsis from spontaneous perforation of pyometra and potentially avoid unnecessary hysterectomy.

## INTRODUCTION

Pyometra is defined as the accumulation of purulent material in the uterine cavity resulting from interference of its natural drainage [1, 2]. It is an uncommon condition with a reported incidence ranging from 0.1% to 0.5% in all gynaecological patients and an incidence approaching 13.6% in elderly patients [2]. Pyometra tends to occur in postmenopausal women and is associated with benign or malignant gynaecological tumours, colorectal tumours, radiation cervicitis, congenital anomalies, puerperal infections and intrauterine devices [1, 3]. The accumulation of purulent material in the uterine cavity and accompanying necrosis of the uterine wall can lead to spontaneous uterine perforation with diffuse peritonitis, a rare complication with a reported incidence of 0.01–0.05% [2, 4]. This report explores a case of spontaneous uterine perforation secondary to pyometra in a patient presenting with sepsis.

## CASE REPORT

A 68-year-old postmenopausal woman presented to the general surgery service at a small rural hospital after being transferred from a peripheral facility. Her past medical history includes previous cerebrovascular accidents with permanent deficits, hypertension, type 2 diabetes mellitus and osteoarthritis. Her initial presentation was significant for abdominal pain of gradual onset, as well as signs of septic shock, including tachycardia, fever, hypotension and leukocytosis with a white blood cell count of 47.8. Generalized peritonitis was noted on examination. A chest X-ray performed prior to transfer revealed free air under the right haemidiaphragm. On arrival at the surgical facility, the patient was seen to have stabilized after the administration of crystalloids. Blood and urine cultures were sent, and broad-spectrum antibiotics were initiated. A urinalysis revealed elevated leukocytes but no other abnormalities. Urine cultures demonstrated colonization with *Escherichia coli* and blood cultures were negative.

**Figure 1:**
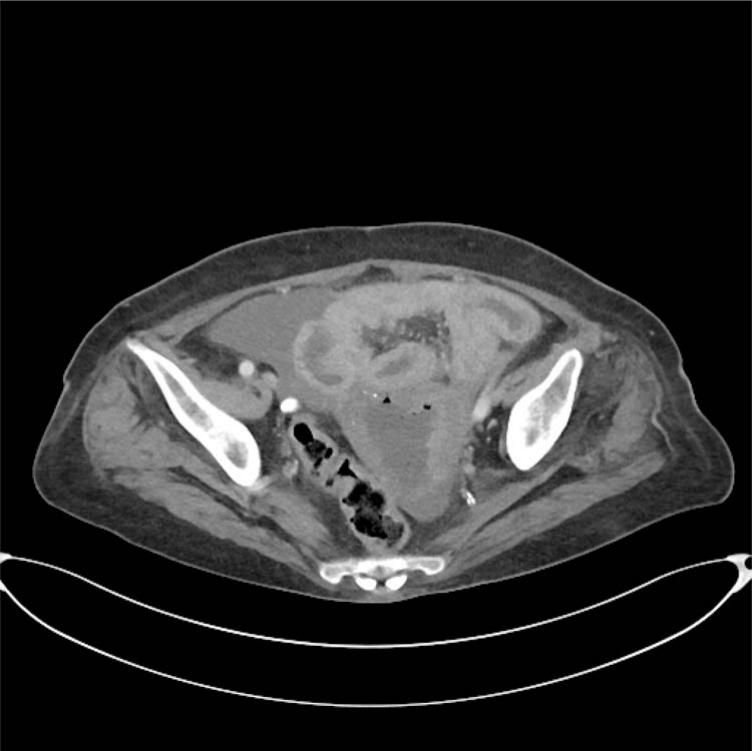
Pyometra with moderate ascites.

**Figure 2:**
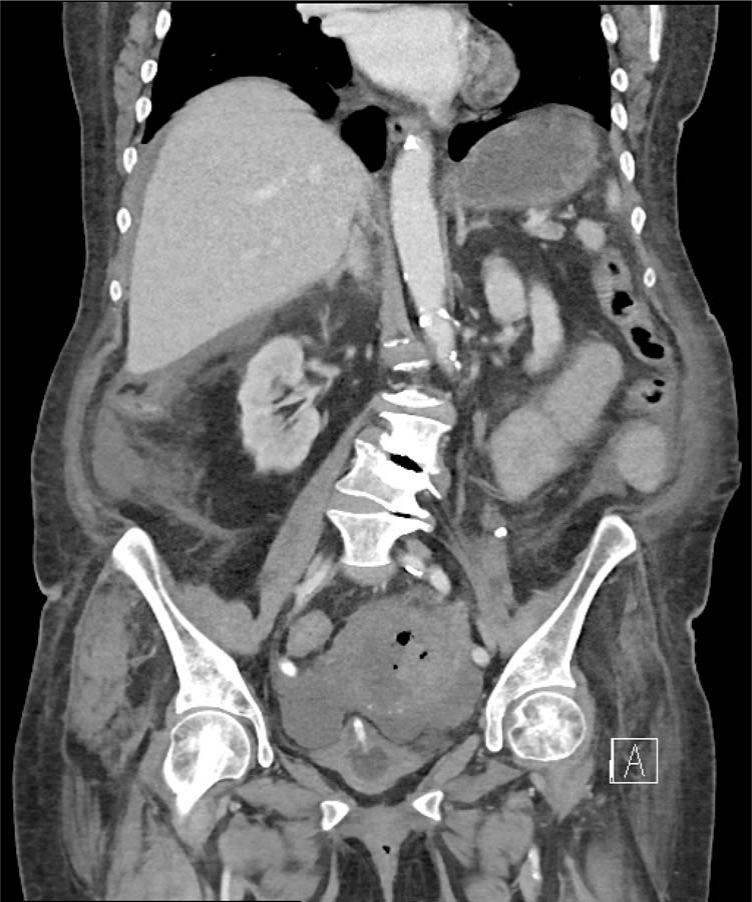
Pneumoperitoneum demonstrated in the perihepatic and perigastric regions.

A computed tomography scan revealed significant abdominal ascites and scattered locules of intraperitoneal free air suggestive of a perforated viscous, although no clear gastrointestinal (GI) source was identified (Figs 1 and 2). In addition, evidence of pyometra with possible necrosis and intramural air within the uterine wall was identified. Emergent laparotomy was performed with complete exploration of the peritoneal cavity. The lesser sac was entered, and complete duodenal kocherization was performed, allowing for visualization of the entire GI tract. No GI perforation was identified. A 1-cm defect was seen on the fundus of the uterus with minimal ischaemic tissue surrounding the defect. Purulent material was seen to be emanating from the uterine cavity. The gynaecology service was consulted, and the uterus was assessed via intraoperative hysteroscopy with uterine lavage. Samples were sent for cytology, including uterine effluent and peritoneal fluid. In addition, uterine tissue at the borders of the defect were excised and sent for histopathologic review. All samples ultimately showed no evidence of malignancy.

Given concerns regarding extensive inflammatory changes in the pelvis and the risk of morbidity associated with hysterectomy in the acute setting, the decision was made, to close the defect and widely drain the pelvis. A heavy absorbable suture was used for primary closure of the uterine defect and two drains were placed. Following closure of the abdomen, the patient was transferred to the intensive care unit and convalesced without complications, ultimately making a full recovery.

## DISCUSSION

Spontaneous perforation of the uterus in the setting of pyometra is often difficult to diagnose clinically, as the symptoms associated with this entity commonly mimic those of GI tract disease [4]. In our case, even with cross-sectional imaging the diagnosis was uncertain, particularly given the extensive intraperitoneal air identified. While a previous case of spontaneous perforation of pyometra diagnosed via dynamic transvaginal ultrasound was identified on literature review, a significant number of reported cases were diagnosed at the time of laparotomy or laparoscopy [1, 4–7].

Traditionally, total abdominal hysterectomy with bilateral salpingo-oophorectomy (TAH + BSO) has been considered the preferred surgical approach in the management of spontaneous perforation of pyometra [8]. However, in the absence of clear evidence of malignancy, the role of either simple hysterectomy or TAH + BSO becomes less clear, particularly in the emergent setting. Some authors have advocated for the use of damage control principles in patients with severe septic shock [9]. Our patient demonstrated signs of severe sepsis at presentation; however, she became stable enough to allow for extensive exploration of both the peritoneal cavity and uterine cavity.

Matsumoto *et al*. [9] described performing a TAH + BSO in a delayed fashion after initially stabilizing their patient with abbreviated surgery. However, in our case histopathology revealed no evidence of malignancy, and as a result hysterectomy was not pursued, thus eliminating the morbidity associated with this procedure. Furthermore, in the era of increasing awareness of the psychosocial impact of emergency hysterectomy on women, the acute care surgeon should perhaps endeavour to preserve the uterus whenever feasible [10, 11].

It is worth highlighting the use of intraoperative hysteroscopy and uterine lavage that proved invaluable in this case. To the author’s knowledge, this technique has not been widely published in the literature with regard to management of spontaneous perforation of pyometra. Access to these tools undoubtedly aided in intraoperative decision making by allowing the uterine cavity to be directly visualized. In the absence of such resources, it is likely that more conservative approaches to management may be necessary, and hysterectomy may have a more well-defined role.

## CONCLUSION

Spontaneous perforation of pyometra is a rare event that is associated with significant morbidity and mortality. In the appropriate setting, repair of the uterine defect with peritoneal lavage and wide drainage may allow for avoidance of hysterectomy and mitigate the morbidity associated with this procedure. Intraoperative hysteroscopy and uterine lavage are indispensable tools in the management of this clinical entity.

## CONFLICT OF INTEREST STATEMENT

None declared.

## References

[1] Yildizhan B , Uyar E, Sişmanoğlu A, Güllüoğlu G, Kavak ZN. Spontaneous perforation of pyometra. Infect Dis Obstet Gynecol 2006;2006:26786. 17093350 10.1155/IDOG/2006/26786 PMC1581463

[2] Emergui Zrihen Y , Obreros Zegarra LP, García Hernández JA. Spontaneous uterine rupture due to pyometra, a case report. Eur J Obstet Gynecol Reprod Biol 2017;217:182–3. 28899567 10.1016/j.ejogrb.2017.08.042

[3] Ou YC , Lan KC, Lin H, Tsai CC, ChangChien CC. Clinical characteristics of perforated pyometra and impending perforation: specific issues in gynecological emergency. J Obstet Gynaecol Res 2010;36:661–6. 20598053 10.1111/j.1447-0756.2010.01184.x

[4] Huang Y , Tian Q. Postmenopausal spontaneous rupture of pyometra: a case report. Medicine (Baltimore) 2018;97:e13659. 30593136 10.1097/MD.0000000000013659 PMC6314776

[5] Malvadkar SM , Malvadkar MS, Domkundwar SV, Mohd S. Spontaneous rupture of pyometra causing peritonitis in elderly female diagnosed on dynamic transvaginal ultrasound. Case Rep Radiol 2016;2016:1738521. 26989549 10.1155/2016/1738521 PMC4775801

[6] Yazawa H , Imaizumi K. Generalized peritonitis secondary to spontaneously perforated pyometra in elderly women:two cases with different clinical courses and surgical approaches and review of the literature. Fukushima J Med Sci 2020;66:53–9. 32281585 10.5387/fms.2019-30 PMC7269882

[7] Ikematsu Y , Kitajima T, Kamohara Y, Inoue K, Maeda J, Amano M, et al. Spontaneous perforated pyometra presenting as pneumoperitoneum. Gynecol Obstet Invest 1996;42:274–6. 8979103 10.1159/000291980

[8] Konishi Y , Kagabu S, Mori K, Kato M. Uterine perforation of pyometra in a cervical cancer: a case report and literature review. J Obstet Gynaecol 2016;36:378–9. 26977976 10.3109/01443615.2015.1072809

[9] Matsumoto R , Kuramoto S, Muronoi T, Oka K, Shimojyo Y, Kidani A, et al. Damage control surgery for spontaneous perforation of pyometra with septic shock: a case report. Acute Med Surg 2021;8:e657. 34026231 10.1002/ams2.657 PMC8133080

[10] Goudarzi F , Khadivzadeh T, Ebadi A, Babazadeh R. Iranian Women's self-concept after hysterectomy: a qualitative study. Iran J Nurs Midwifery Res 2021;26:230–7. 34277374 10.4103/ijnmr.IJNMR_146_20 PMC8262543

[11] Elmir R , Schmied V, Wilkes L, Jackson D. Separation, failure and temporary relinquishment: women's experiences of early mothering in the context of emergency hysterectomy. J Clin Nurs 2012;21:1119–27. 22176681 10.1111/j.1365-2702.2011.03913.x

